# Translation Control by *p53*

**DOI:** 10.3390/cancers10050133

**Published:** 2018-05-05

**Authors:** Justina Kasteri, Dibash Das, Xuelin Zhong, Leah Persaud, Ashleigh Francis, Hilal Muharam, Moira Sauane

**Affiliations:** 1 Department of Biological Sciences, Herbert H. Lehman College, City University of New York, 250 Bedford Park Boulevard West, Bronx, NY 10468, USA; JUSTINA.KASTERI@lc.cuny.edu (J.K.); dd791@hunter.cuny.edu (D.D.); xzhong@gradcenter.cuny.edu (X.Z.); LEAH.PERSAUD@lehman.cuny.edu (L.P.); ashleighfrancis64@gmail.com (A.F.); HILAL.MUHARAM@lc.cuny.edu (H.M.); 2 Department of Biology, The Graduate Center, City University of New York, 365 Fifth Avenue, Room, 250 Bedford Park Boulevard West, Bronx, NY 10468, USA

**Keywords:** *p53*, eukaryotic initiation factor 4F complex, ternary complex, translation regulation, the mammalian target of rapamycin, Casein Kinase 2

## Abstract

The translation of mRNAs plays a critical role in the regulation of gene expression and therefore, in the regulation of cell proliferation, differentiation and apoptosis. Unrestricted initiation of translation causes malignant transformation and plays a key role in the maintenance and progression of cancers. Translation initiation is regulated by the ternary complex and the eukaryotic initiation factor 4F (eIF4F) complex. The *p53* tumor suppressor protein is the most well studied mammalian transcription factor that mediates a variety of anti-proliferative processes. Post-transcriptional mechanisms of gene expression in general and those of translation in particular play a major role in shaping the protein composition of the cell. The *p53* protein regulates transcription and controls eIF4F, the ternary complex and the synthesis of ribosomal components, including the down-regulation of rRNA genes. In summary, the induction of *p53* regulates protein synthesis and translational control to inhibit cell growth.

## 1. Introduction

**Figure 1:**
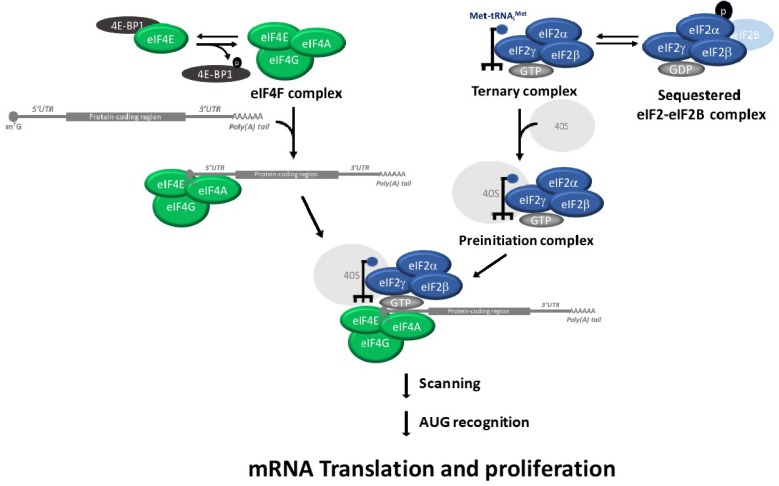
Scheme showing the eukaryotic translation initiation pathway.

Translation initiation plays a critical role in the regulation of cell proliferation, differentiation and apoptosis (reviewed in [1]). Translation initiation is regulated by the assembly of ternary complex and eIF4F complexes [2]. The eukaryotic Initiation factor 2 (eIF2) and the initiating methionyl-tRNA (Met-tRNAi) form the ternary complex. The ternary complex recruits the 40S ribosomal subunit to form the 43S pre-initiation complex. The 43S pre-initiation complex binds to the mRNA cap with the participation of other translation initiation factors, such as the eIF4F complex. The pre-initiation complex scans the 5′ untranslated region (5′ UTR) of the mRNA for the initiator AUG codon, which is the location where the 60S ribosomal subunit joins to form the 80S ribosome. An important point of translation initiation regulation is the guanosine diphosphate (GDP)/guanosine triphosphate (GTP) exchange of eIF2, which is catalyzed by eIF2B in order to initiation a new round of translation. This GDP–GTP exchange of eIF2 is inhibited when the alpha subunit of eIF2 (eIF2α) is phosphorylated on S51 [3]. The eIF2α phosphorylation results in a reduction in the overall rate of translation initiation [4]. Phosphorylated eIF2α binds with high affinity to the guanine nucleotide exchange factor eIF2B and thereby inhibits the exchange of the GDP for GTP in eIF2 (Figure 1) [5,6,7]. The inhibitory effects of the ternary complex reduce the expression of oncogenic proteins and increases the expression of tumor-suppressor and pro-apoptotic proteins [8,9]. 

Although most mRNAs are recruited to the ribosome via the recognition of their 5′ end cap structure (m^7^GpppN, where N can be any nucleotide), a subset of mRNAs can be translated using an internal ribosome entry site (IRES) [2]. As mentioned, the translation initiation is regulated by the ternary complex and eIF4F assembly [10]. The eIF4F is a complex comprised of eIF4A (an RNA helicase), eIF4E (the cap-binding subunit) and eIF4G (a large scaffolding protein for eIF4A, which is called eIF4E). The eIF4F complex binds to the cap structure, unwinds the complex secondary structures of the mRNA 5′ UTRs template and recruits the 43S pre-initiation complex. The eIF4F complex stimulates the recruitment of ribosomes to the mRNA template. eIF4A stimulates translation of both capped and uncapped mRNAs in vitro [10].

A key regulatory step of translation is the initiation stage, which involves the recruitment of ribosomes to the 5′ UTR of the mRNA. The eIF4E-binding proteins (consists of three members: 4E-BP1, 4E-BP2 and 4E-BP3) are repressors of eIF4F. Hypo-phosphorylated 4E-BPs binds to eIF4E and prevents recruitment of the translation machinery to mRNA. In contrast, 4E-BP hyper-phosphorylation causes disruption of the 4E-BP:eIF4E complex and renders eIF4E available for eIF4F formation, which results in an abrogation of eIF4E-binding activity (Figure 1) [11]. Similarly, the tumor suppressor protein programmed cell death protein 4 regulates the protein translation by preventing eIF4A from interacting with eIF4G, leading to translational suppression [12].

The *p53* tumor suppressor protein is the most well studied mammalian transcription factor that mediates a variety of anti-proliferative processes [13]. Elucidating the effect of *p53* on translation initiation in the context of cell cycle regulation is essential in understanding the role that mutations or deregulation of *p53* play in cancer biology. The post-transcriptional mechanisms of gene expression in general, especially those of translation, play a major role in shaping the protein composition of the cell [14]. The *p53* protein regulates transcription and also controls ribosome biogenesis and eukaryotic initiation factors. In this review article, we examine the role of *p53* as a regulator of the ternary complex, the eIF4F complex and translation ribosome biogenesis.

## 2. *p53* Restricts the Ribosome Biogenesis

Ribosomes are responsible for transferring the information contained in mRNAs to proteins. Ribosome biogenesis takes place in the nucleolus [15,16]. The ribosomal DNA (rDNA) genes are organized at the nucleolar organizer regions [17,18]. The human 60S ribosome subunit is a complex molecule composed of three ribosomal RNA (5S, 5.8S and 28S rRNA) and 47 distinct ribosomal proteins (RPs) [19]. This complex is responsible for peptide bond formation and quality control of nascent peptides [20]. The human 40S ribosome subunit, which is responsible for unwinding and scanning mRNAs, is also a complex molecule composed of one strand of 18S ribosomal RNA (rRNA) and 33 distinct RPs. Both experimental models and clinical studies indicate that disturbances in the ribosomal biosynthesis and/or protein synthesis have been shown to play a major role in tumorigenesis [21,22,23]. 

**Figure 2:**
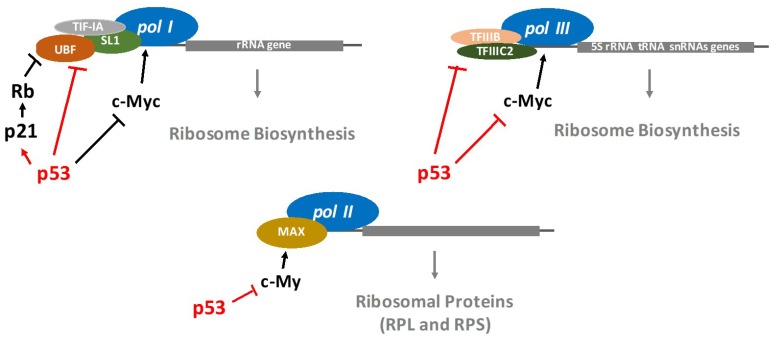
Key mechanism by which *p53* inhibits RNA polymerases.

It is well established that *p53* restricts the ribosome biogenesis*.* Indeed, the regulation of RNA polymerases (*pol*) by *p53* has been extensively studied. RNA *pol* I synthesizes ribosomal RNAs (except for 5S rRNA) [24]. RNA *pol* II synthesizes mRNA precursors as well as most small nuclear RNA and microRNAs, while RNA *pol* III manufactures transfer RNA (tRNAs), the 5S rRNA as well as other small RNAs involved in RNA processing and transport. The *p53*-mediated RNA *pol* I transcriptional repression involves *p53* interfering with a set of proteins required for the assembly and initiation of transcriptional machinery on the rRNA promoter [25]. Interestingly, Zhai et al. demonstrated that *p53*-mediated growth suppression occurred by repressing rRNA gene transcription [26]. Specifically, *p53* directly binds to the TATA (the Hogness box)-binding protein (TBP) and TBP-associated factors, disrupting their interaction with upstream binding factors and thereby repressing RNA *pol* I transcription. It has also been reported that *p53* is able to activate the transcription of RNA *pol* I and II through repression of c-Myc expression [27] (Figure 2). A novel, selective RNA *pol* I transcription inhibitor, CX-5461 (Cylene Pharmaceuticals/Senhwa Biosciences), promotes cancer-specific activation of *p53* and apoptosis in malignant B cells [28], which is an interesting development in the search for new anti-cancer therapies.

**Figure 3:**
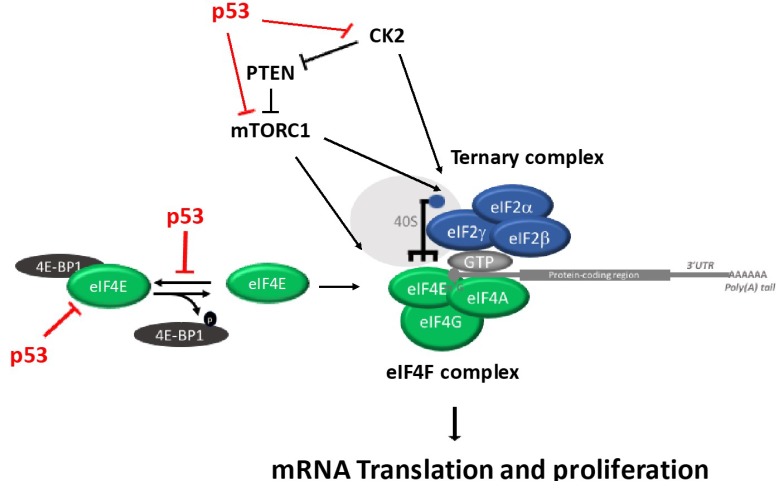
Schematic diagram outlining the regulation of eIF4F and Ternary Complex and the different steps known to be modulated by *p53*.

Furthermore, *p53* inhibits RNA pol III activity, which generates tRNAs, the 5S rRNA as well as other small RNAs involved in RNA processing and transport. Indeed, the wild-type *p53* directly interacts with the TBP-containing general factor (TFIIIB), which prevents the attachment of RNA *pol* III onto DNA [29,30,31] (Figure 3).

## 3. *p53* Regulates the Transcription of RP Genes

Newly synthesized ribosomal proteins (RPs) are imported into the nucleolus from the cytosol. In response to nucleolar stress, several RPs (RPL5, RPL11, RPL23, RPS3, RPS7) as well as 5.8S and 5S rRNA translocate from the nucleolus to the nucleoplasm. This subset of nucleolar elements binds to and inhibits the activity of murine double minute 2 (MDM2), resulting in *p53*-mediated cell cycle arrest and apoptosis [32]. Cancer cells that harbor mutant *p53* (R248W) leads to an up-regulation of L37, P1 and S2 expression [33]. Interestingly, in response to DNA damage, the ribosomal protein RPL26 binds to both 5′-UTR and 3′-UTR of *p53* mRNA, enhancing *p53* translation and leading to *p53*-dependent and MDM2-independent cell cycle arrest [34,35]. Upon genotoxic stress, *p53* directly induces the expression of the ribosomal protein S27-like (RPS27L), thus positively regulating p21 protein expression. This ultimately leads to p21-mediated cell cycle arrest [36,37].

*p53* not only regulates rRNA transcription, but also controls the processing of pre-rRNAs. Fibrillarin (FBL) is a nucleolar protein vital for methylation and processing of pre-rRNAs [38]. Marcel et al. demonstrated that *p53* inhibits FBL expression levels. The *p53*-dependent FBL inhibition leads to a high translational fidelity (i.e., nonsense suppression or amino acid misincorporation) and increases the initiation of internal ribosome entry site (IRES)-dependent translation [39]. Understanding the role of *p53* in pre-RNAs processing has the potential to identify future therapeutic targets on the ribosome population with altered rRNA methylation patterns.

## 4. p53 Regulates Ternary Complex and eIF4F Assembly Regulation

It remains largely unknown how the two major regulatory branches that regulate translation, ternary complex and eIF4F complex are coordinated. Interestingly, Gandin et al. demonstrated a coordinated regulation of eIF2α and eIF4E via CK2 and mTORC1 [40]. The mTOR (a phosphatidylinositol 3-kinase) forms functionally distinct complexes, including: mTOR complex 1 (mTORC1) and mTOR complex 2 (mTORC2). The mTORC1 regulates mRNA translation, protein synthesis and cell growth, while mTORC2 regulates cellular metabolism, survival and the cytoskeletal organization. Protein kinase casein kinase 2 (CK2) belongs to the serine/threonine protein kinase family and consists of two catalytic (alpha and/or alpha’) subunits and two regulatory (beta) subunits [41,42]. CK2 regulates key cell growth and survival pathways, including translation regulation and DNA damage response pathways [42].

A 50% reduction of global protein synthesis was determined in vitro utilizing thermosensitive murine erythroleukemic cells carrying wild-type *p53* [43]. Specifically, it was demonstrated that the inhibition of the ribosomal protein S6 kinase (p70S6k), which is the upstream kinase of 4E-BP1, by p53 strongly attenuated the global protein synthesis [43].

Recent data showed that TRIM22, a *p53* target gene, inhibits the binding of eIF4E to eIF4G [44]. Furthermore, *p53* activation causes dephosphorylation and cleavage of the initiation factor eIF4GI and the eIF4E-binding protein 4E-BP1 [45,46]. Interestingly, Petroulakis et al. demonstrated that the *p53* controls the 4E-BP-dependent senescence and transformation. Specifically, *p53*-deficient mice have an increased risk of tumorigenesis in the absence of both 4E-BP1 and 4E-BP2. Conversely, primary fibroblasts expressing *p53*, but missing 4E-BPs, undergo premature senescence and are resistant to transformation. These findings indicate that the combined effect of absence of 4E-BPs and p53 loss synergistically enhanced cell proliferation and tumorigenesis, which provides new insights into anticancer drug therapy [47]. *p53* inhibits protein synthesis through directly inhibiting the expression of eIF4E [48] and inhibiting the eIF4F complex assembly by enhancing the de-phosphorylation of 4E-BP1 [45]. Although *p53* does not directly interact with components of the ternary complex, it controls translation by interacting with several components of the eIF4F complex. Specifically, Rahman and colleagues showed that *p53* is not able to control the ternary complex pathway through PKR-eIF2α activation [49]. Instead, the *p53* inhibits mTOR signaling [50] and CK2 protein kinase activity (Figure 3).

The eukaryotic translation factor 5A (eIF5A) promotes the elongation of translation [51]. Posttranslational hypusine modification of eIF5A by deoxyhypusine synthase (DHS) and deoxyhypusine hydroxylase (DOHH) regulates eIF5A activity [51]. Preukschas et al. demonstrated that targeting eIF5A with a specific DHS-inhibitor has an antiproliferative effect in glioblastoma cell lines, while sparing the normal human astrocytes. Furthermore, targeting eIF5A results in *p53*-mediated premature senescence in glioblastoma cell lines [52].

As mentioned, CK2 and mTORC1 coordinate the activation of the ternary complex and elF4F complex. The phosphorylation of elF2B by CK2 increases the ternary complex assembly, which also up-regulates 4E-BP phosphorylation through mTORC1. It has been shown that there is phosphorylation of *p53* in a highly-conserved residue (S392) by CK2 [53,54]. Catrogiovanni and colleagues showed that the phosphorylation at residue S392 regulates *p53* mitochondrial translocation and transcription-independent apoptosis after cell exposure to genotoxic agents. Furthermore, the regulatory beta subunit of CK2 can interact with *p53* and this interaction reduces the DNA binding activity and the transactivation function of *p53* [55,56]. Conversely, the wild-type *p53* interacts with and inhibits CK2 protein kinase activity [57]. As described above, the role of CK2 and mTORC1 are major regulators of translation [40]. The exact mechanism by which the interaction between CK2, mTOR and *p53* regulates translation awaits further studies.

## 5. Genome-Wide Analysis

To investigate the effects of *p53* on the genome-wide translational regulation, Zaccara et al. used the polysome profiling technique [58]. The authors identified about 340 genes whose translation is regulated by *p53* [59]. Loayza-Puch et al. combined RNA sequencing and ribosomal profiling analyses in order to methodically discover transcriptional and translational control in cells under the following conditions: quiescence, senescence, normal proliferation and neoplastic transformation. The authors demonstrated that the global repression of protein synthesis is mediated by *p53* activation and consequent mTOR inhibition. The authors conclude that transcriptional regulation mediates cell-cycle arrest, while the global translation inhibition impacts cell growth [60].

## 6. Conclusions

*p53* is associated with numerous signaling pathways, which are involved in the regulation of cellular responses to stress. Its effect on growth arrest or programmed cell death is context-dependent: both in terms of cellular type and of physiological state. It is therefore not surprising that translation regulation is critically affected by *p53*. A better understanding of these intricate pathways and the complex interplay between *p53* and various signaling pathways that regulate translation and ribosome synthesis could open up novel strategies for cancer diagnosis, prevention and *p53*-based therapies.
